# First De Novo Transcriptome of the Copepod *Rhincalanus gigas* from Antarctic Waters

**DOI:** 10.3390/biology9110410

**Published:** 2020-11-23

**Authors:** Chiara Lauritano, Vittoria Roncalli, Luca Ambrosino, Matthew C. Cieslak, Adrianna Ianora

**Affiliations:** 1 Marine Biotechnology Department, Stazione Zoologica Anton Dohrn, Villa Comunale, 80121 Napoli, Italy; ianora@szn.it; 2 Integrative Marine Ecology Department, Stazione Zoologica Anton Dohrn, Villa Comunale, 80121 Napoli, Italy; vittoria.roncalli@szn.it; 3 Research Infrastructure for Marine Biological Resources Department, Stazione Zoologica Anton Dohrn, Villa Comunale, 80121 Napoli, Italy; luca.ambrosino@szn.it; 4 Pacific Biosciences Research Center, University of Hawai’i at Manoa, 1993 East-West Rd., Honolulu, HI 96822, USA; dechtmatt@gmail.com

**Keywords:** copepod, transcriptome, South Shetland Trench, Antarctic waters

## Abstract

**Simple Summary:**

Compared to more accessible sites, organisms inhabiting Antarctic waters have been poorly investigated. This study provides the first molecular resource (transcriptome from whole individual) for the eucalanoid copepod *Rhincalanus gigas*, one of the predominant zooplankton species of Antarctic waters. Sequence analyses identified possible adaptation strategies adopted by the organism to cope with cold environments. Among those, we identified in *R. gigas* transcriptome three predicted genes encoding for antifreeze proteins and gene duplication within the glutathione metabolism pathway. This new molecular resource, provided here, will be useful to study the physiology, ecology, and biology of *R. gigas* and it increases the information available for polar environments.

**Abstract:**

Antarctic waters are the largest almost untapped diversified resource of our planet. Molecular resources for Antarctic organisms are very limited and mostly represented by sequences used for species genotyping. In this study, we present the first transcriptome for the copepod *Rhincalanus gigas*, one of the predominant zooplankton species of Antarctic waters. This transcriptome represents also the first molecular resource for an eucalanoid copepod. The transcriptome is of high quality and completeness. The presence of three predicted genes encoding antifreeze proteins and gene duplication within the glutathione metabolism pathway are suggested as possible adaptations to cope with this harsh environment. The *R. gigas* transcriptome represents a powerful new resource for investigating the molecular basis associated with polar biological processes and ecology.

## 1. Introduction

Polar regions represent the largest almost untapped diversified resource of our planet. Antarctic waters can be considered an extreme environment where, in order to survive, organisms have evolved adaptations to cope with seasonal changes in day length, food availability, and ice cover, as well as to consistently cold waters [1].

In the Southern Ocean, zooplankton communities are dominated by copepods in terms of numbers and biomass [2]. These tiny organisms play an essential role in carbon cycling between primary producers and higher trophic consumers. In the last decade, reductions in coastal ice and sea ice, as well as iceberg scour increases, have been observed along the Antarctic Peninsula as a result of global atmospheric warming. Increased production of melting water and consequent changes in sea-ice dynamics represent an extreme challenge for marine biota which has adapted for millions of years to the region’s extreme conditions [1]. Questions have been raised on how copepods distributions will be affected and how organisms will physiologically adapt to these changes. Because of difficulties and costs in their sampling, species living in such extreme environments have not been well studied, and molecular resources for Antarctic zooplankton species are scarce. To date, there are only three available resources for Antarctic zooplankters: (1) the harpacticoid copepod *Tigriopus kingsejongensis* [3], (2) the krill (*Euphausia superba*) (KrillDB) [4] and (3) the pteropod *Limacina helicina* [5].

*Rhincalanus gigas* (Brady, 1883) is a dominant species in Antarctic and sub-Antarctic circumpolar waters [6]. This copepod is most abundant in the area from sub-Antarctic waters to the north of the Polar Front [6,7], with low seasonal abundance fluctuations probably due to reduced metabolic rates [8,9]. *R. gigas* is an epipelagic copepod, predominantly feeding on small-particle phytoplankton, mainly diatoms, and protozoans [10,11]. The life cycle of *R. gigas* is still unclear. The copepod seems to have a flexible two year cycle depending on conditions, and it is not clear if, during winter, the copepod enters a period of developmental arrest (diapause) [6,9,12]. Recent investigations on the succession of micro- and meso-zooplankton during a warm annual cycle (December 2010–December 2011) in an Antarctic coastal environment (Potter Cove) reported *R. gigas* as the most abundant representative zooplankton taxon [13]. Thus, based on its key role, this copepod can be used as a model organism for investigations of zooplankton physiological ecology. Here, using high-throughput sequencing, we generated a de novo transcriptome for *R. gigas* adult individuals. This reference transcriptome is also the first molecular resource for an eucalanoid copepod. The *R. gigas* transcriptome is a new source that can provide the basis for gene discovery or for gene expression studies to investigate changes in response to environmental stressors and to identify the molecular mechanisms of environmental adaptation to extreme environments.

## 2. Materials and Methods

### 2.1. Ethics Statement

The collection of marine samples from the South Shetland Trough was approved by the Foreign and Commonwealth office. The permit (No. 51/2015 and 52/2015) was granted under Section 3, Section 6, and Section 7 of the Antarctic Act 1994 to Alan J. Jamieson (the permit holder), expedition Chief, on behalf of the University of Aberdeen. The main purpose of the permit was to collect the marine organisms from deep and cold habitats with the goal to survey the zooplankton composition of the South Shetland Trough and to compare and contrast the fauna with other trench ecosystems.

### 2.2. Zooplankton Sampling

*Rhincalanus gigas* individuals were collected on 21 and 22 December 2015, from two stations of the South Shetland Trench (SST) (Station 1: latitude −58.0703, longitude −61.326945; Station 2: latitude −58.199624, longitude −60.958174) during the “PharmaDEEP” cruise (https://www.eurofleets.eu/access/previous-calls/eurofleets2-regional-2-call-results/eurofleets2-funded-project-pharmadeep-results/) [14]. Zooplankton was collected at 100 m depth with a Nansen net (200 μm mesh, 1 m diameter) towed vertically. Collections were immediately diluted with 0.22 μm filtered seawater and adult *R. gigas* were live sorted; six individuals were immediately preserved in RNAlater Stabilization Reagent (Thermo Fisher Scientific, Waltham, MA, USA).

### 2.3. DNA Extraction and Genotyping

Total DNA was extracted from individual *R. gigas* adults using Trizol according to the manufacturer’s protocol (Invitrogen, Thermo Fisher Scientific, Waltham, MA, USA). DNA quantity was assessed by Nano-Drop (ND-1000 UV-Vis spectrophotometer; NanoDrop Technologies, Wilmington, NC, USA). The mitochondrial cytochrome oxidase subunit I region (COI) (fragment 518 bp) was amplified using the following primers: COI-F (5′–GGCCAAAACAGGGAGAGATA–3′) and COI-R (5′–CGGGACTCAGTATAATTATTCGTCTA–3′) [15]. Reactions were carried out in a 20 µL volume with 2 µL of 10× PCR reaction buffer Roche (Sigma-Aldrich, Saint Louis, MO, USA), 2 µL of 0.1% Bovine Serum Albumin (BSA), 2 µL of 10× 2 mM dNTP, 0.8 µL of 5 U/mL Taq Roche, 1 µL of 20 pmol/µL for each oligo, 1.5 µL of template DNA, and nuclease-free water. The PCR program consisted of a denaturation step at 94 °C for 3 min, 35 cycles at 94 °C for 1 min, 50 °C for 45 s, and 72 °C for 1 min, and a final extension step at 72 °C for 7 min. Amplified PCR products were analyzed by 1.5% agarose gel electrophoresis in TBE (Tris/Borate/EDTA) buffer. The resulting bands were excised from the gel and extracted according to GenElute^TM^ Gel Extraction Kit (Sigma-Aldrich, Saint Louis, MO, USA), and the fragments were sequenced. Sequence reactions were obtained by using the BigDye Terminator Cycle Sequencing technology (Applied Biosystems, Thermo Fisher Scientific, Waltham, MA, USA) in automation, using the Agencourt CleanSEQ Dye Terminator Removal Kit (Agencourt Bioscience, Beckman Coulter, Brea, CA, USA) and a Robotic Station Biomek FX (Beckman Coulter, Brea, CA, USA). Products were analyzed on an Automated Capillary Electrophoresis Sequencer 3730 DNA Analyzer (Applied Biosystems, Thermo Fisher Scientific, Waltham, MA, USA). To confirm their identity, sequences were BLASTed (using the Basic Local Alignment Search Tool, BLAST) against the National Center for Biotechnology Information (NCBI) nucleotide database (nr/nt) by using the BLASTn algorithm.

### 2.4. RNA Extraction

Total RNA was extracted from six *R. gigas* individuals using Trizol according to the manufacturer’s protocol (Invitrogen). Each individual was transferred in 500 µL of Trizol (Invitrogen) and was homogenized using the Tissuelyser (MM300, Retsch, Conquer Scientific, San Diego, CA, USA) and tungsten carbide beads (3 mm) (Qiagen, Valencia, CA) for 5 min (2 min followed by 3 min, without removing the samples from the instrument) at 20.1 Hz until it was completely homogenized [16]. After centrifuging at 12,000 rpm for 10 min at 4 °C to remove debris, the supernatant was passed approximately five times through a 0.1 mm syringe needle (as in [17]). After an initial quality assessment (Nanodrop), each sample was treated with DNase I (Invitrogen) following the manufacturer’s instructions to remove hypothetical DNA contamination. Lastly, the quality of total RNA was checked using an Agilent 2100 Bioanalyzer (Agilent Technologies, Santa Clara, CA, USA).

### 2.5. RNA Sequencing

Two *R. gigas* individuals, confirmed from the genotyping analysis (mtCOI 98.8% identity), were processed for RNA-Seq. Total RNA samples were shipped on dry ice to the Genomix4life S.R.L. (Baronissi, Salerno, Italy). There, using the TruSeq Stranded mRNA Sample Prep Kit (Illumina, San Diego, CA, USA), complementary DNA (cDNA) libraries were prepared with 500 ng/each of purified RNA. Libraries were quantified using the TapeStatiFon 4200 (Agilent Technologies) and Invitrogen Qubit fluorometer (Thermo Fisher Scientific), and then pooled so that each index-tagged sample was present in equimolar amounts, with a final concentration of the pooled samples of 2 nM. Each sample was indexed and paired-end sequenced (PE 75 bp) using an Illumina NextSeq 500 System (Illumina) on a single lane.

### 2.6. De Novo Assembly and Functional Annotation

The quality of the two RNA-Seq libraries was assessed using FASTQC (Babraham bioinformatics; https://www.bioinformatics.babraham.ac.uk/index.html). From each library, Illumina adapters (TruSeqLT universal primer), the first 12 bp from each read, reads < 50 bp long, and reads with an average Phred score < 30 were removed using Trimmomatic (v.2.0.0) [18]. From each library, an 8% average of low-quality reads were removed, and more than 30 million reads were left for downstream analyses. A reference transcriptome was generated combining the two quality filtered libraries that were de novo assembled using Trinity software (v. 2.0.6) [19] at the National Center for Genome Analysis Support’s (NCGAS; Indiana University, Bloomington, IN, USA) Mason Linux cluster. The initial parameters of Trinity were set as follows: --seqType fq --CPU 32 --max_memory 200G -- min_contig_length 300 -- normalize_max_read_cov 50. Summary of the statistics was obtained using the script TrinityStats.pl (v2.0.6). The completeness of the de novo assembly was assessed by mapping the quality-filtered reads to the reference transcriptome using Bowtie2 software (default setting: v2.1.0) [20]. Additionally, BUSCO software (v1.22) [21] was used to identify highly conserved genes among eukaryote (“core genes”) which are indicative of completeness. This analysis was performed using the Arthropoda dataset consisting of 2675 single-copy orthologs.

Raw reads were submitted to the National Center of Biotechnology Information (NCBI) to the Sequence Read Archive under the Bioproject PRJNA639356.

TransDecoder software (v5.3.0) (https://github.com/TransDecoder/TransDecoder/releases) (default settings) was used to identify protein coding sequences. These predicted proteins were functionally annotated using InterProScan software (v5.33) [22] (Supplementary Table S1). An additional functional annotation was obtained by performing a sequence similarity search of the obtained protein collection against SwissProt protein database [23] using BLASTp [24] with a maximum E-value threshold of 10^−3^ (https://www.uniprot.org/ updated at April 2020). The assembly and annotation statistics of the *R. gigas* transcriptome were compared with those from other calanoid copepods. Transcriptomes for *Calanus finmarchicus* [25], *Neocalanus flemingeri* [26], and *Labidocera madurae* [27] were selected based on their high quality according to the following criteria: (1) RNASeq with Illumina platforms; (2) a minimum of 10 million high-quality reads per library; and (3) publicly available assembly annotation statistics. 

On the basis of functional hypotheses of genes and pathways involved in cold adaptation, we investigated the glutathione metabolism pathway and antifreeze proteins. From the Kyoto Encyclopedia of Genes and Genomes (KEGG)web-accessible resource [28], the fruit fly *Drosophila melanogaster* was used as a reference organism to identify the “key” enzymes in the glutathione metabolism pathway (map00480). The *D. melanogaster* “expected” enzymes were searched based on their annotation (Enzyme commission number) in the *R. gigas* annotated transcriptome. Similarly, the *R. gigas* reference transcriptome was also searched for antifreeze proteins (AFP). The identified *R. gigas* transcript encoding AFP proteins were then checked for the presence of the characteristic C-type lectin (CTL) or carbohydrate-recognition (CRD) (CLECT) domains using SMART program [29] and aligned with their top BLAST hits. Additionally, the transcriptome was in silico mined using the NCBI BLAST algorithm for two known AFPs from the copepod *Stephos longipes* (NCBI Accession no. ACL00837 and ACL00838) [30]. However, for both queries, this search did not result in any *R. gigas* homologs.

## 3. Results

### 3.1. Genotyping

Two of the six genotyped individuals were confirmed as *R. gigas*. For both individuals, mtCOI sequences were 98.8% identical (nucleotide) to the deposited *R. gigas* mtCOI sequence on NCBI (GenBank Acc. n. JN663356.1).

### 3.2. De Novo Assembled Reference Transcriptome

The *R. gigas* de novo assembly, generated by combining two RNA-seq libraries, resulted in 78,285 transcripts with an average length of 877 bp, a maximum of 10,033 bp, and an N50 value of 1143 bp (Table 1). The majority (60%) of the 31,851 “Trinity predicted genes” were singletons. The remaining 12,411 genes possessed 2 to 44 isoforms. Completeness of the reference transcriptome was confirmed by the high mapping rate of the libraries against the reference. The mapping rate for both samples against the transcriptome averaged to 82% with 44% mapped more than one time (Table 1). BUSCO analysis identified 70% of the complete ortholog genes, with only 12% being fragmented (Table 1).

### 3.3. Functional Annotation

**Figure 1:**
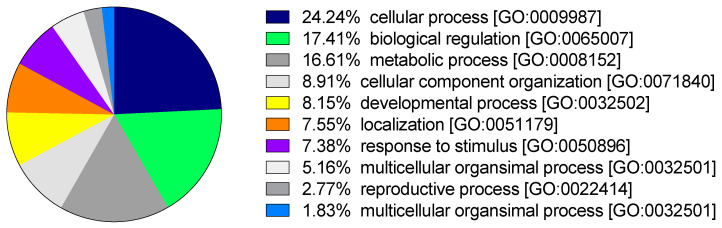
*Rhincalanus gigas* reference transcriptome annotation against Gene Ontology (GO) database. Pie chart showing the percentage of annotated transcripts within each GO term belonging to the Gene Ontology biological process (BP) category.

Eighty percent of the transcripts (61,983) had coding regions, with 55% of these being successfully functionally annotated by scanning the SwissProt database. Over 39% of these transcripts also had assigned Gene Ontology (GO) terms, and ca. 49% of them were annotated among protein families (PFAM database) (Supplementary Table S1). Transcripts annotated within the gene ontology “biological process” (BP) category covered broad conserved eukaryotic processes with cellular process (GO:0009987), biological regulation (GO:0065007), and metabolic process (GO:0008152) as the top three represented GO terms (Figure 1).

The assembly and annotation statistics suggest that the *R. gigas* transcriptome is of at least similar quality and depth to the other calanoid references (Table 2). For each assembly, the N50 was >1000 bp and the self-mapping rate was >80% (Table 2). Differences in the number of assembled transcripts might be related to differences in the version of the Trinity software and on its settings. BUSCO analysis resulted in a coverage of 70–90% of the complete “core” genes for all the assemblies. The annotation statistics, as the number of transcripts annotated against SwissProt and Gene Ontology database, suggest that the *R. gigas* transcriptome is of at least similar quality and depth to these others, being most similar to the *N. flemingeri* transcriptome (Table 2).

Pathway annotation against the KEGG database resulted in 1623 transcripts that were associated with 118 different pathways. Among the top 10 represented KEGG pathways, seven were associated with the “metabolism” category, two were associated with “environmental information processing”, and a single pathway was associated with “genetic information processing” (Table 3). The metabolism category included pathways involved with nucleotide (e.g., purine), amino acids (e.g., glutathione), and carbohydrate (e.g., glycolysis/gluconeogenesis) metabolism. The phosphatidylinositol 3′-kinase (PI3K)-Akt signaling and mTOR signaling pathways, part of the “environmental information processing” category, were involved in signal transduction. Digestion of dietary carbohydrate (R-HSA-189085) and protein ubiquitination (PWY-7511) were another two pathways that were highly represented from the annotation of the transcriptome against the Reactome and Metacyc databases. The complete list of annotated pathways is available in Supplementary Table S2.

### 3.4. Adaptation to Cold: Glutathione Metabolism and Antifreeze Proteins

Glutathione metabolism is the fourth most represented pathway in *R. gigas* transcriptome (Table 3); this pathway is complete (all *D. melanogaster* “expected” enzymes) with all enzymes being full-length (Supplementary Table S2). Most enzymes were encoded by different “predicted genes”, with some multiple variants (isoforms), most likely derived from alternative splicing, as well as single-nucleotide polymorphisms. All enzymes were annotated (SwissProt) with E value > 1 × 10^−40^, and transcripts encoding for different predicted genes had different top BLAST hits.

According to the KEGG hierarchy, the pathway consisted of three networks: (1) Glutathione biosynthesis (N00899), (2) glutathione reduction (N00904), and (3) nicotinamide-adenine-dinucleotide phosphate (NADP^+^) reduction (N00905) (Supplementary Table S2). The glutathione biosynthesis network includes five enzymes annotated as 5-oxoprolinase (EC 3.5.2.9), gamma-glutamylcysteine synthetase (EC 6.3.2.3), glutathione synthase (EC 6.3.2.3), phospholipid-hydroperoxide glutathione peroxidase (EC 1.11.1.12), and glutathione peroxidase (EC 1.11.1.9). Three enzymes (EC 3.5.2.9.5, EC 6.3.2.2, EC 6.3.2.2) were represented by a single “predicted gene”. The glutathione synthase enzyme (EC 6.3.2.3) was represented by two predicted genes, while the glutathione peroxidase enzyme (EC 1.11.1.9) was encoded by five different predicted genes (Supplementary Table S2). One of these five predicted genes was a singleton and encoded a complete protein, while the remaining four had multiple isoforms with some partially encoding proteins (Supplementary Table S2). The second network included a single enzyme glutathione reductase (EC 1.8.1.7), which corresponded in *R. gigas* to a single predicted gene encoding a complete protein (Supplementary Table S2). The last network, NADP^+^ reduction, included three enzymes: isocitrate dehydrogenase (EC 1.1.1.42), 6-phosphogluconate dehydrogenase (EC 1.1.1.44), and glucose-6-phosphate 1-dehydrogenase (EC 1.1.1.49). Two enzymes (EC 1.1.1.42 and EC 1.1.1.44) were represented by two *R. gigas* predicted genes, while the 6-phosphogluconate dehydrogenase enzyme (EC 1.1.1.44) was represented by three predicted genes.

**Figure 2:**
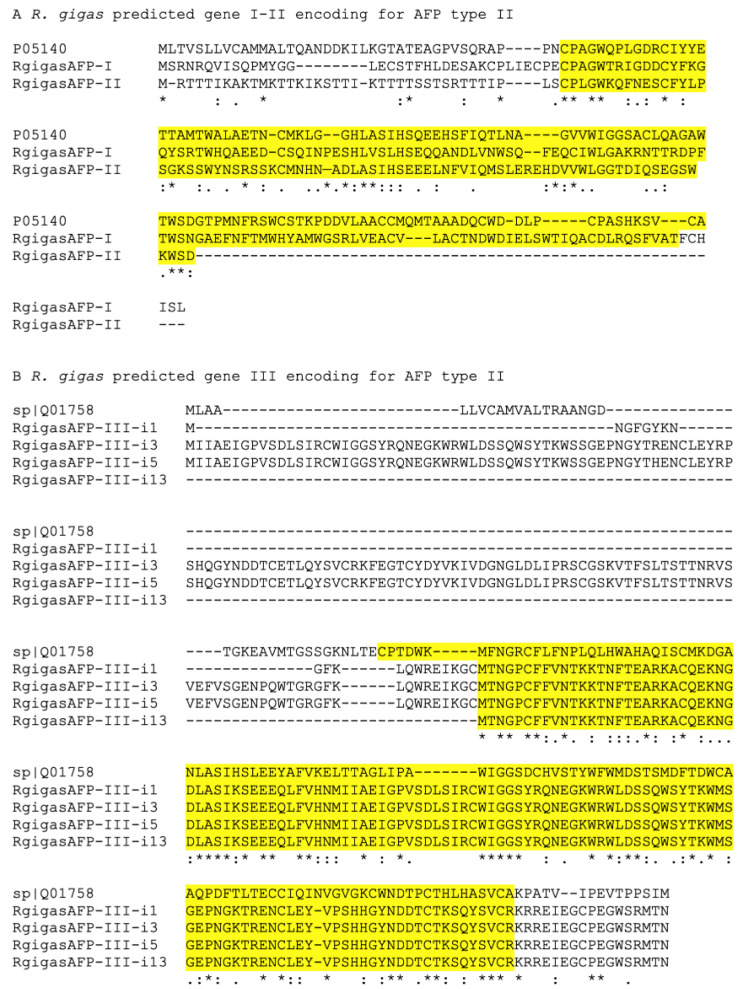
Alignment of three antifreeze protein (AFP) sequences predicted from the *R. gigas* de novo transcriptome. Three genes were predicted (I–III) on the basis of automated annotation. (**A**) Alignment of the first two sequences (*R. gigas* AFP I and II) with their top BLAST hit, AFP type II from *Hemitripterus americanus* (P05140). (**B**) Alignment of the third *R. gigas* predicted gene, AFP III, with its top BLAST hit AFP type II from *Osmerus mordax* (Q01758). The *R. gigas* AFP III consisted of multiple isoforms (i1, i3, i5, and i13). In yellow, the C-type lectin carbohydrate-recognition domain typical of AFP type II is highlighted.

Three predicted genes encoding antifreeze proteins (ASPs) were identified in the *R. gigas* transcriptome (Supplementary Table S3). All predicted genes (AFP I–III) had highest homology with the fish AFP known as type II, which likely evolved from calcium dependent (c-type) lectins. Type II AFPs are usually found in sea raven, smelt, and herring, consistent with *R. gigas* AFP I and II being highly similar to AFP from the sea raven *Hemitripterus americanus* and AFP III being similar to AFP from rainbow smelt *Osmerus mordax* (Supplementary Table S3) (Figure 2A,B). AFP III had multiple isoforms which all encoded identical proteins (Figure 2B). All *R. gigas* AFPs contained the typical C-type lectin carbohydrate-recognition domain of AFPs type II (Figure 2). Additional mining of the *R. gigas* transcriptome for AFPs from the Antarctic sea ice copepod *Stephos longipes* did not generate any significant hit. This negative result is likely associated with the fact that those isoforms had no homology with any antifreeze proteins from the metazoan linage [30].

## 4. Discussion

The number of high-quality transcriptomes, especially for copepods, is still limited, although, in recent years, there has been an increase in molecular resources for metazoan zooplankton [31,32,33,34,35,36,37]. In this study, we present the first molecular resource for an eucalanoid copepod, *Rhincalanus gigas*. This copepod is one of the most abundant zooplankters of Antarctic waters and could be used as a future model organism for investigations of zooplankton physiological ecology in extreme environments. Due to its completeness, the *R. gigas* transcriptome is a good reference for gene discovery and for gene expression studies to investigate the biology, physiology, and ecology of this copepod.

Organisms that have to cope with harsh conditions (e.g., freezing temperatures, limited food availability, and sea-ice coverage) require a specific and complex reprogramming at the transcriptional, proteomic, and metabolic levels which alter communication and signaling between subcellular organelles [38]. Temperature is one of the main factors driving organism adaptation [39]. Adaptations to cold might involve specific rearrangement (e.g., gene duplication) of genes associated with metabolic pathways. Enrichment of enzymes involved in purine, pyrimidine, cysteine, and methionine metabolism, as well as in glycolysis and gluconeogenesis, has been reported in two yeast strains both having specific adaptation to temperature; *Saccharomyces kudriavzevii* (CA111) is a cryotolerant strain, while *S. cerevisiae* (96.2) is a thermotolerant strain [40]. The authors also reported defects in lipid metabolism, oxidoreductase, and vitamin pathways that impair yeast fitness at cold temperatures in both strains. Their results suggest that temperature-induced redox imbalances could be compensated for by increased glycerol accumulation or the production of cytosolic acetaldehyde through the deletion of glycerol-3-phosphate dehydrogenase (GUT2) or alcohol dehydrogenase (ADH3), respectively [40]. Recently, Enriquez and Colinet [41] also reported that insects remodeled various processes in order to cope with the cold, including purine metabolism and aminoacyl transfer RNA (tRNA) biosynthesis. In the harpacticoids *Tigriopus kingsejongensis* (Antarctic) and *T. japonicus*, significant functional divergence of genes associated with energy metabolism, purine/pyrimidine metabolism, pentose and glucoronate interconversions, glycerolphospholipid metabolism, and oxidative phosphorylation pathways was reported [3]. Enrichment in the Antarctic copepod suggests a potential role of these pathways in the adaptation to the colder environment. Here, we report that, in the *R. gigas* transcriptome, several metabolic pathways such as purine/pyrimidine metabolism were among the top ten represented pathways, consistent with what is known for other organisms adapted to cold environments.

In marine organisms, response to oxidative stress plays a fundamental role in the maintenance of cellular homeostasis. Reactive species, especially reactive oxygen species (ROS), can alter cellular homeostasis by damaging DNA, RNA, proteins, lipids, and carbohydrates, which, in worst cases, can even induce organism death [42,43]. Glutathione is an important cell scavenger which is involved in radical compound deactivation. It is synthetized by glutathione synthase and mostly found in its reduced and oxidized state. The oxidized state can be converted again to the reduced state via the enzyme glutathione reductase (GR), rendering the thiol group of the cysteinyl residue again available as a source of one reducing equivalent [42]. During oxidative stress, glutathione plays a key role in protection and detoxification as a cofactor of glutathione peroxidases and glutathione-*S*-transferases (GSTs) [44]. It also synergistically interacts with other components of the antioxidant defense system such as vitamin C, vitamin E, and superoxide dismutase. The stressful conditions of extreme environments such as Antarctic waters induce, in most eukaryotes, an increase in the production of reactive oxygen species (ROS). In the Antarctic notothenioid *Dissostichus mawsoni*, duplication of genes involved in the antioxidant process is considered as a possible strategy to compensate for the highest vulnerability to ROS production and oxidative damage (reviewed in [45]).

The glutathione metabolism pathway in *R. gigas* was the fourth represented pathway. The completeness of this pathway was demonstrated by the presence of all expected enzymes in *D. melanogaster*, mostly encoding for full-length proteins. Evidence of possible gene duplication for several enzymes within this pathway was found, which is consistent with what has been reported for *D. mawsoni*. However, further investigations on gene multiplicity are required.

Antifreeze glycoproteins (AGFPs) are a class of proteins which are considered an evolutionary adaptation allowing organisms to survive at subzero temperatures [46,47]. AFPs which inhibit the growth and recrystallization of ice, binding to small ice crystals [46], have been found in many marine organisms including bacteria, crustaceans, and microalgae. Two putative AFPs have been identified in the sea-ice Antarctic copepod *Stephos longipes* [30]. Both isoforms possess thermal hysteresis activity and showed high homology with diatoms and bacteria but no homology with any metazoan lineage, suggesting horizontal gene transfer (HGT) [30]. *R. gigas* possesses three predicted genes encoding antifreeze proteins, all similar to fish type II AFPs. Furthermore, the *R. gigas* AFPs are highly different from the sea ice copepod isoforms, confirming that these latter proteins had no homology with metazoans. This is the first evidence that AFPs are also present in copepods that do not live in ice. However, further investigations are needed in order to assess the activity of these *R. gigas* proteins.

## 5. Conclusions

In the last decade, the application of high-throughput sequencing has become more common for developing molecular resources for non-model species, including those of ecological relevance. Transcriptomics approaches used in gene discovery and gene expression studies can be used to investigate the biology and ecophysiology of key organisms. However, both types of studies depend on high-quality reference transcriptomes. In this study, we presented the first molecular resource for an eucalanoid copepod, *Rhincalanus gigas*, one of the most abundant zooplankters of Antarctic waters. Our data show gene duplication within the glutathione metabolism pathway, as well as the presence of three predicted genes encoding antifreeze proteins suggesting, possible adaptation mechanisms to cope with this extreme environment. According to its high quality and depth, the *R. gigas* transcriptome is a powerful new resource for investigating the ecophysiology of this copepod within the context of life history strategies and ecosystem dynamics.

## Figures and Tables

**Table 1:** Summary of assembly statistics for *Rhincalanus gigas* reference transcriptome. De novo assembly was generated by combining two RNA-Seq library using Trinity software. Mapping percentage refers to the average of the two individuals’ libraries mapped back to the reference transcriptome. BUSCO analysis was performed using the Arthropoda dataset consisting of 2675 single-copy orthologs.

*R. gigas* Reference Transcriptome	
Trinity Transcripts (*n*)	78,285
Trinity predicted genes (*n*)	31,851
Minimum length (bp)	301
Maximum length (bp)	10,033
Average contig length (bp)	877
GC content (%)	40.52
N50 (bp)	1143
N25 (bp)	2089
N75 (bp)	612
**Mapping**	
Overall mapping (%)	81.8
Mapping >1 time (%)	44
**BUSCO Eukaryotic Genes**	
Complete (%)	70
Fragmented (%)	12
Missing (%)	22

**Table 2:** Comparison of *Rhincalanus gigas* transcriptome with other copepod de novo assemblies. *R. gigas* (current study) assembly and annotation statistics were compared with those from *Calanus finmarchicus* [25], *Neocalanus flemingeri* [26] and *Labidocera madurae* [27]. For each assembly, information on sequencing, assembly, and functional annotation steps are provided.

	*R. gigas*	*C. finmarchicus*	*N. flemingeri*	*L. madurae*
Sequencing	Illumina NextSeq	Illumina HiSeq	Illumina NextSeq	Illumina NextSeq
NCBI BioProject	PRJNA639356	PRJNA236528	PRJNA324453	PRJNA324849
**De novo assembly**				
Transcripts (n)	78,285	206,041	140,841	211,002
Minimum length (bp)	301	301	301	301
Maximum length (bp)	10,033	23,068	24,981	23,836
N50	1143	1418	1452	1184
Overall self-mapping (%)	82	89	92	90.8
**Functional annotation**				
Transcripts with coding region (n)	61,983 (79.1%)	np	108,092 (76.7%)	72,391 (32%)
Transcripts with BLAST hits (n)	34,238	28,616	62,126	62,980
Transcripts with GO terms (n)	24,426	10,334	59,544	60,097
**BUSCO**				
Complete (%)	70	79	79	76
Fragmented (%)	12	8	6	11
Missing (%)	22	12	15	12

NCBI, National Center for Biotechnology Information; np, not provided in the study.

**Table 3:** Top 10 KEGG pathways in the *Rhincalanus gigas* transcriptome. For each pathway, name, entry, and class are reported according to the BRITE hierarchy in the KEGG database (https://www.genome.jp/kegg/brite.html). For each pathway, the number of transcripts annotated as enzymes is listed.

Pathway Name	Entry	Class	Transcripts
Purine metabolism	00230	Nucleotide metabolism (metabolism)	210
Cysteine and methionine metabolism	00270	Amino-acid metabolism (metabolism)	128
Pyrimidine metabolism	00240	Nucleotide metabolism (metabolism)	121
Glutathione metabolism	00480	Other amino-acid metabolism (metabolism)	116
Starch and sucrose metabolism	00500	Carbohydrate metabolism (metabolism)	115
mTOR signaling pathway	04150	Signal transduction (environmental information processing)	115
Glycolysis/Gluconeogenesis	00010	Carbohydrate metabolism (metabolism)	106
Aminoacyl transfer RNA (tRNA) biosynthesis	00970	Translation (genetic information processing)	100
Amino sugar and nucleotide sugar metabolism	00520	Carbohydrate metabolism (metabolism)	99
PI3K/Akt signaling pathway	04151	Signal transduction (environmental information processing)	97

## Supplementary Materials

The following are available online at https://www.mdpi.com/2079-7737/9/11/410/s1: Table S1. Summary of functional annotation for *R. gigas* reference transcriptome. Results of automated annotation of *R. gigas* transcripts coding protein (*n* = 61,983) against several databases. For each database, name, ID, and database description are listed. Three databases (PANTHER, HMAP SFLD, and TIGRFAM) for which the annotation was <3% are not included in the table; Table S2. List of *R. gigas* transcripts encoding enzymes involved in the glutathione metabolism pathway. Transcripts have been annotated against KEGG pathway database and represent the “expected” enzymes using *D. melanogaster* as reference organism. Enzymes are ordered following the KEGG hierarchy (three networks). In each network, the *R. gigas* transcripts, with protein length (description and bp), annotation E value, and top blast hit (NCBI Accession number) are listed; Table S3. List of the *R. gigas* transcripts encoding antifreeze proteins (AFPs). Transcripts have been annotated against SwissProtein database. For each *R. gigas* transcript, transcript ID, protein length (description and bp), annotation E value, and top BLAST hit (NCBI Accession number, organism) are listed.
