# Supplementary material for: First De Novo Transcriptome of the Copepod Rhincalanus gigas from Antarctic Waters
Source: Biology (Basel). 2020 Nov 23;9(11):410. doi: 10.3390/biology9110410 (PMC7700397; doi:10.3390/biology9110410)
Supplement: Supplementary file 1 [file biology-09-00410-s001.pdf]

Supplementary materials

Table S1. Summary of functional annotation for R. gigas reference transcriptome. Results of automated annotation of R. gigas transcripts coding protein (n=61,983) against several databases. For each database, name and ID and database description are listed. Three databases (PANTHER, HMAP SFLD and TIGRFAM) for which the annotation was <3% are not included in the Table**.**

Database name (ID)	Database description	Annotation (%)
SwissProt	Protein database	55.2
INTERPRO	Protein sequence analysis & classification	57.8
GO	Gene Ontology	39.4
SUPERFAMILY	Structural and functional annotation for all proteins and genomes	46.3
PRINTS	Protein families	83
PFAM	Protein families	49.4
CDD	Protein conserved domain	16.9
GENE3	CATH domain families from 3D-structures	48.2
SMART	Protein domain	23.4
PATHWAYS	Metabolic pathways from KEGG, REACTOME and METACYC databases	9.2
EC NUMBER	KEGG Enzyme commission number	18.6
PROSITEPATTERNS	Protein database using patterns as motif descriptors	13.2
PROSITEPROFILES	Protein database using profiles as motif descriptors	29.6

Table S2: List of R. gigas transcripts encoding enzymes involved in the glutathione metabolism pathway. Transcripts have been annotated against KEGG pathway database and represent the "expected" enzymes using D. melanogaster as reference organism. Enzymes are ordered following the KEGG hierarchy (3 networks). In each network, the R. gigas transcripts, with protein length (description and bp), annotation E value and top blast hit (NCBI Accession number) are listed**.**

	Protein length (complete, I=internal, P=partial)	Protein length (bp)	Evalue annotation	Topblast Accesion #	
**Network 1: GSH biosynthesis**					
**3.5.2.9-5-Oxoprolinase**					
TRINITY_DN18491_c0_g1_i1	complete	1282	0.0	O14841	OPLA_HUMAN
TRINITY_DN18491_c0_g1_i2	complete	850	0.0	P97608	OPLA_RAT
TRINITY_DN18491_c0_g1_i3	complete	1282	0.0	O14841	OPLA_HUMAN
**6.3.2.2-Gamma-glutamylcysteine synthetase**					
TRINITY_DN21557_c0_g1_i1	internal	553	0.0	P48506	GSH1_HUMAN
TRINITY_DN21557_c1_g1_i1	complete	636	0.0	P48506	GSH1_HUMAN
**6.3.2.3-Glutathione synthetase (GSH synthetase)**					
TRINITY_DN14939_c0_g1_i1	prime_partial	495	2.77e-119	P35668	GSHB_XENLA
TRINITY_DN19928_c0_g2_i1	complete	502	1.31e-129	P35668	GSHB_XENLA
TRINITY_DN19928_c0_g2_i2	complete	520	2.40e-125	P35668	GSHB_XENLA
**1.11.1.12-Phospholipid hydroperoxide glutathione peroxidase**					
TRINITY_DN18436_c1_g2_i1	complete	129	1.05e-34	Q9N2J2	GPX4_BOVIN
TRINITY_DN18436_c1_g2_i3	complete	114	5.29e-41	Q9N2J2	GPX4_BOVIN
TRINITY_DN18436_c1_g2_i4	complete	120	1.00e-39	Q9N2J2	GPX4_BOVIN
TRINITY_DN18436_c1_g2_i7	complete	114	1.74e-39	Q9N2J2	GPX4_BOVIN
**1.11.1.9-Glutathione peroxidase**					
TRINITY_DN19336_c0_g1_i10	complete	106	9.25e-18	G9JJU2	GPX_PROCL
TRINITY_DN19336_c0_g1_i4	5prime_partial	127	7.13e-22	G9JJU2	GPX_PROCL
TRINITY_DN19336_c0_g1_i5	complete	106	9.25e-18	G9JJU2	GPX_PROCL

TRINITY_DN19336_c0_g1_i6	complete	106	2.84e-18	G9JJU2	GPX_PROCL
TRINITY_DN19336_c0_g1_i7	complete	106	1.48e-17	G9JJU2	GPX_PROCL
TRINITY_DN19336_c0_g1_i9	5prime_partial	127	5.68e-24	G9JJU2	GPX_PROCL
TRINITY_DN19336_c0_g1_i3	5prime_partial	147	2.06e-25	G9JJU2	GPX_PROCL
TRINITY_DN21001_c0_g1_i1	complete	107	9.02e-19	G9JJU2	GPX_PROCL
TRINITY_DN21001_c0_g1_i10	complete	108	9.02e-19	G9JJU2	GPX_PROCL
TRINITY_DN21001_c0_g1_i12	complete	108	5.89e-19	G9JJU2	GPX_PROCL
TRINITY_DN21001_c0_g1_i3	complete	108	5.89e-19	G9JJU2	GPX_PROCL
TRINITY_DN21001_c0_g1_i4	complete	108	5.89e-19	G9JJU2	GPX_PROCL
TRINITY_DN21001_c0_g1_i6	complete	108	5.29e-19	G9JJU2	GPX_PROCL
TRINITY_DN21001_c0_g1_i7	complete	108	5.29e-19	G9JJU2	GPX_PROCL
TRINITY_DN21001_c0_g1_i8	5prime_partial	108	9.45e-19	G9JJU2	GPX_PROCL
TRINITY_DN21001_c0_g1_i9	5prime_partial	158	1.45e-38	G9JJU2	GPX_PROCL
TRINITY_DN19460_c0_g1_i1	complete	147	2.50e-41	Q4AEI2	GPX1_HYLLA
TRINITY_DN19460_c0_g1_i4	complete	140	1.06e-55	Q4AEI2	GPX1_HYLLA
TRINITY_DN19460_c0_g1_i5	complete	147	2.29e-41	Q4AEI2	GPX1_HYLLA
TRINITY_DN19460_c0_g1_i6	3prime_partial	113	1.74e-43	Q4AEI2	GPX1_HYLLA
TRINITY_DN19460_c0_g1_i8	complete	140	7.34e-57	Q4AEI2	GPX1_HYLLA
TRINITY_DN19460_c0_g1_i9	complete	140	1.02e-55	Q4AEI2	GPX1_HYLLA
TRINITY_DN15942_c0_g1_i1	complete	147	2.18e-49	P11352	GPX1_MOUSE
TRINITY_DN14552_c0_g1_i4	complete	140	7.38e-54	A6QLY2	GPX7_BOVIN
**Network 2:Glutathione reduction**					
**1.8.1.7-Glutathione reductase(GR)**					
TRINITY_DN19043_c0_g1_i1	complete	507	2.28e-173	P47791	GSHR_MOUSE
**Network 3:NADP+ reduction**					
**1.1.1.42-Isocitrate dehydrogenase [NADP]**					
TRINITY_DN22501_c0_g1_i1	complete	737	0.0	P16100	IDH_AZOVI
TRINITY_DN22501_c0_g1_i2	internal	112	1.97e-33	P50216	IDH_CORGL
TRINITY_DN22667_c0_g1_i1	complete	759	0.0	P16100	IDH_AZOVI
TRINITY_DN22667_c0_g1_i3	3prime_partial	582	0.0	P41561	IDH2_COLMA
TRINITY_DN22667_c0_g1_i2	3prime_partial	416	4.19e-147	P16100	IDH_AZOVI

**1.1.1.44-6-phosphogluconate dehydrogenase**					
TRINITY_DN14414_c0_g1_i1	3prime_partial	103	1.86e-51	Q9DCD0	6PGD_MOUSE
TRINITY_DN15592_c0_g1_i1	internal	119	1.09e-61	P00349	6PGD_SHEEP
TRINITY_DN15592_c0_g1_i2	internal	112	9.87e-56	P00349	6PGD_SHEEP
TRINITY_DN15592_c0_g1_i3	internal	119	3.13e-61	P00349	6PGD_SHEEP
TRINITY_DN15592_c0_g1_i4	internal	112	1.08e-54	P00349	6PGD_SHEEP
TRINITY_DN15592_c0_g1_i5	internal	119	6.97e-61	P00349	6PGD_SHEEP
TRINITY_DN23631_c3_g1_i1	internal	183	2.93e-85	Q17761	6PGD_CAEEL
TRINITY_DN23631_c3_g1_i2	complete	487	0.0	P52209	6PGD_HUMAN
TRINITY_DN23631_c3_g1_i3	complete	487	0.0	P52209	6PGD_HUMAN
**1.1.1.49-Glucose-6-phosphate 1-dehydrogenase (G6PD)**					
TRINITY_DN21382_c0_g3_i2	internal	184	1.37e-96	Q27638	G6PD_DROYA
TRINITY_DN7346_c0_g1_i1	internal	117	1.17e-48	Q27638	G6PD_DROYA
TRINITY_DN21382_c0_g1_i1	internal	466	0.0	P12646	G6PD_DROME
TRINITY_DN21382_c0_g1_i2	internal	424	0.0	P12646	G6PD_DROME
TRINITY_DN21382_c0_g3_i1	5prime_partial	525	0.0	P12646	G6PD_DROME
TRINITY_DN21382_c0_g3_i3	5prime_partial	525	0.0	P12646	G6PD_DROME

Table S3. List of the R. gigas transcripts encoding antifreeze proteins (AFPs). Transcripts have been annotated against SwissProtein database. For each R. gigas transcript, transcript ID, protein length (description and bp), annotation E value and top blast hit (NCBI Accession number, organism) are listed.

Transcripts encoding R. gigas antifreeze proteins	Annotation	Protein length (complete, internal, partial)	Protein length (bp)	Evalue annotation	Topblast Accesion #	
TRINITY_DN19051_c0_g1_i1	Type-2 ice-structuring protein (Type II antifreeze protein) (AFP)	5prime_partial	207	2.25e-09	P05140	*Hemitripterus americanus*
TRINITY_DN20104_c0_g1_i1	Type-2 ice-structuring protein (Type II antifreeze protein) (AFP)	internal	229	9.87e-16	Q01758	*Osmerus mordax*
TRINITY_DN20104_c0_g1_i13	Type-2 ice-structuring protein (Type II antifreeze protein) (AFP)	3prime_partial	141	1.08e-16	Q01758	*Osmerus mordax*
TRINITY_DN20104_c0_g1_i3	Type-2 ice-structuring protein (Type II antifreeze protein) (AFP)	internal	240	4.73e-15	Q01758	*Osmerus mordax*
TRINITY_DN20104_c0_g1_i5	Type-2 ice-structuring protein (Type II antifreeze protein) (AFP)	internal	290	5.40e-15	Q01758	*Osmerus mordax*
TRINITY_DN21643_c1_g1_i1	Type-2 ice-structuring protein (Type II	internal	184	6.83e-12	P05140	*Hemitripterus americanus*

	antifreeze protein) (AFP)					
TRINITY_DN21643_c1_g1_i3	Type-2 ice-structuring protein (Type II antifreeze protein) (AFP)	internal	163	1.04e-12	P05140	*Hemitripterus americanus*
